# Perioperative Chemotherapy on Survival in Patients With Upper Urinary Tract Urothelial Carcinoma Undergoing Nephroureterectomy: A Population-Based Study

**DOI:** 10.3389/fonc.2020.00481

**Published:** 2020-04-21

**Authors:** Ting-Shuai Zhai, Liang Jin, Li-Ming Feng, Zhen Zhou, Xiang Liu, Huan Liu, Wei-Guo Ma, Jing-Yi Lu, Wei Chen, Xu-Dong Yao, Lin Ye

**Affiliations:** ^1^Department of Urology, Shanghai Tenth People's Hospital, Tongji University School of Medicine, Shanghai, China; ^2^Department of Urology, Shawan People's Hospital, Tacheng, China; ^3^Department of Urology, First Clinical Medical College, Nanjing Medical University, Nanjing, China; ^4^Department of Urology, Shanghai Putuo District People's Hospital, Tongji University School of Medicine, Shanghai, China; ^5^Department of Urology, Karamay Central Hospital, Karamay, China; ^6^Department of Urology, Tongxin People's Hospital, Wuzhong, China

**Keywords:** chemotherapy, neoplasm staging, Surveillance, Epidemiology and End Results (SEER) program, survival analysis, upper urinary tract urothelial carcinoma

## Abstract

**Objectives:** To estimate the stage-specific impact of perioperative chemotherapy on survival for upper urinary tract urothelial carcinoma (UTUC) patients treated with nephroureterectomy (NU).

**Methods:** Overall, 7,278 UTUC patients treated with NU from 2004 to 2015 were identified within the SEER database. Kaplan–Meier plots were used to elucidate overall survival (OS) and cancer-specific survival (CSS) rates. Multivariable Cox regression analyses were used to test the impact of chemotherapy on survival rates, after stratifying according to pathological stage.

**Results:** Chemotherapy was performed in 17.3% of patients and in 5.7, 11.5, 25.4, and 51.3% of patients with, respectively, pT1, pT2, pT3, and pT4 disease (*P* < 0.001). In multivariable analyses, perioperative chemotherapy was associated with a lower OS in pT2 patients and a lower CSS in pT1 disease (both *P* < 0.05), while predisposed to a higher OS in pT3 and pT4 patients (both *P* < 0.01). Moreover, perioperative chemotherapy was prone to a higher OS or CSS in pN+ disease compared to no chemotherapy (both *P* < 0.01).

**Conclusion:** Perioperative chemotherapy was more frequently performed in locally advanced UTUC patients. The beneficial effect of chemotherapy on OS was evident in pT3/pT4 and pN+ patients. In addition, a clear CSS benefit was observed in patients who received chemotherapy for pN+ UTUC, while perioperative chemotherapy may reduce CSS for pT1 and OS for pT2 patients following NU.

## Introduction

Upper urinary tract urothelial carcinomas (UTUCs), accounting for only 5–10% of urothelial carcinomas (UCs), are rare malignancies arising from the renal pelvis or ureters with different anatomic and biological attributes from lower tract urothelial malignancies (1, 2). Unlike bladder cancer, UTUC requires different therapeutic strategies, which may be due to the anatomic and biological differences (3). Nephroureterectomy (NU) is the accepted surgical management of high-risk UTUC, while recurrences are common even after NU (2). Therefore, it is reasonable to consider perioperative chemotherapy in an effort to decrease recurrence risk. Over the past decades, several researches have tested the effect of perioperative chemotherapy on the oncologic outcomes. However, the role of perioperative chemotherapy for patients treated with NU remains controversial. A cohort study of patients fit enough to receive systemic chemotherapy for metastatic UTUC reported an overall survival (OS) benefit to combine chemotherapy and NU (4), while a recent multi-center study found that adjuvant chemotherapy after NU did not improve OS compared to observation (5). This may result from the dilemma to determine which types of patients are suitable for perioperative chemotherapy. The most frequent adverse reaction of cisplatin-based regimen is nephrotoxicity (6), which may significantly reduce survival in patients with postoperative renal dysfunction (7, 8).

Renal function, comorbidities, tumor location, grade, and stage, and molecular marker status should be taken into account when determining the optimal treatment regimen for UTUC patients (9). To date, no previous study has tested the impact of perioperative chemotherapy on stage-specific survival following NU for UTUC patients. To resolve this issue, we examined the effect of perioperative chemotherapy on OS and cancer-specific survival (CSS). Our hypothesis stated that perioperative chemotherapy might benefit both OS and CSS, which is consistent across all tumor stages.

## Materials and Methods

### Study Population

UTUC patients (7,278), which have histologically confirmed transitional cell carcinoma of the renal pelvis or ureter who underwent NU, were selected within the Surveillance, Epidemiology and End Results (SEER) database from 2004 to 2015. All of them were non-metastatic transitional cell carcinoma located within the renal pelvis or ureter. Patients were excluded if tumor stage, tumor grade, and lymph node dissection (LND) status were unclear.

### Definition of Variables for Analyses

Patients were stratified according to the presence or absence of perioperative chemotherapy including both neoadjuvant chemotherapy (NC) and adjuvant chemotherapy (AC). Covariates consisted of age at diagnosis, gender (male, female), race (white, other), marital status (married, unmarried, unknown), primary site (renal pelvis, ureter), laterality (left, right, paired), tumor size (≤ 2 and >2 cm), tumor stage (T1, T2, T3, T4), lymph node stage (N0, Nx, N1–3), tumor grade (I, II, III, IV), and year of surgery (2004–2007, 2008–2011, 2012–2015).

### Statistical Analysis

Continuous variables are reported as mean ± s.d. and were analyzed by Student's *t*-test. Categorical variables were compared using χ^2^ test or Fisher's exact test, as appropriate. Kaplan–Meier plots graphically depicted OS and CSS curves. Our Cox regression analyses comprised two steps. In the first step, Cox regression analyses tested the impact of perioperative chemotherapy (Yes vs. No/Unknown) on OS and CSS. In the second step, Cox regression analyses examined the effect of lymph node stage (pN0 vs. pNx vs. pN1–3) on OS and CSS. The patient population was stratified into node-negative (N0), node-positive (N1–3), and regional lymph nodes not removed (Nx) groups. In all multivariable analyses, covariates consisted of age, gender (male vs. female), race (white vs. other), tumor location (renal pelvis vs. ureter), laterality (left vs. right), tumor size (≤ 2 vs. >2 cm), pathological tumor stage (pT1 vs. pT2 vs. pT3 vs. pT4), pathological lymph node stage (pN0 vs. pNx vs. pN1–3) (AJCC 6th ed.), tumor grade (grade I vs. grade II vs. grade III vs. grade IV), and year of surgery categories (2004–2007 vs. 2008–2011 vs. 2012–2015). Finally, all the aforementioned analyses were repeated for each tumor stage and lymph node stage. The 95% CIs were calculated, and *P* < 0.05 was considered statistically significant. SPSS (IBM SPSS Statistics 25) was used for analyses.

## Results

### General Characteristics

Overall, 7,278 patients (median age 73 years, range: 22–101) underwent NU for UTUC within the SEER database (Table 1). The majority were male (59.0%), of white race (88.2%), have married status (60.7%), had renal pelvis location (69.1%), had left laterality (50.4%), and had big tumor size (73.7%). Overall, 292 patients harbored grade I (4.0%) vs. 1,102 grade II (15.1%) vs. 2,096 grade III (28.8%) vs. 3,788 grade IV (52.0%), and 2,279 patients harbored T1 (31.3%) vs. 1,353 T2 (18.6%) vs. 3,075 T3 (42.3%) vs. 571 T4 (7.8%) stage. Patients, 1,296 (17.8%), were confirmed with pN0 by LND vs. 665 (9.1%) pN1-3, and 5,317 (73.1%) patients were categorized as pNx due to the absence of LND.

**Table 1:** Characteristics for UTUC patients stratified by chemotherapy recode.

	Total^*^ (%)	Chemotherapy recode^*^ (%) LND^*^		***P-*value^‡^**
**Characteristic**		**No/Unknown**	**Yes**	
Total	7,278 (100)	6,020 (82.7)	1,258 (17.3)	
Age (years)^§^				<0.001
Mean ± SD	71.9 ± 10.8	72.8 ± 10.7	67.6 ± 10.1	
Median	73	74	68	
Range	22–101	22–101	31–92	
Gender				0.325
Male	4,295 (59.0)	3,537 (48.6)	758 (10.4)	
Female	2,983 (41.0)	2,483 (34.1)	500 (6.9)	
Race				0.005
White	6,419 (88.2)	5,339 (73.4)	1,080 (14.8)	
Other	859 (11.8)	681 (9.4)	178 (2.4)	
Marital status				<0.001
Married	4,421 (60.7)	3,560 (48.9)	861 (11.8)	
Unmarried	2,588 (35.6)	2,240 (30.8)	348 (4.8)	
Unknown	269 (3.7)	220 (3.0)	49 (0.7)	
Primary site				0.111
Renal pelvis	5,032 (69.1)	4,186 (57.5)	846 (11.6)	
Ureter	2,246 (30.9)	1,834 (25.2)	412 (5.7)	
Laterality				0.064
Left	3,665 (50.4)	3,002 (41.2)	663 (9.1)	
Right	3,607 (49.6)	3,012 (41.4)	595 (8.2)	
Paired	6 (<1%)	6 (<1%)	0 (<1%)	
Tumor size				0.137
≤ 2 cm	1,178 (16.2)	998 (13.7)	180 (2.5)	
>2 cm	5,357 (73.7)	4,409 (60.7)	948 (13.0)	
Unknown	743 (10.1)	613 (8.4)	130 (1.8)	
Grade				<0.001
I	292 (4.0)	278 (3.8)	14 (0.2)	
II	1,102 (15.1)	1,031 (14.2)	71 (1.0)	
III	2,096 (28.8)	1,719 (23.6)	377 (5.2)	
IV	3,788 (52.0)	2,992 (41.1)	796 (10.9)	
T stage				<0.001
T1	2,279 (31.3)	2,149 (29.5)	130 (1.8)	
T2	1,353 (18.6)	1,197 (16.4)	156 (2.1)	
T3	3,075 (42.3)	2,293 (31.5)	782 (10.7)	
T4	571 (7.8)	381 (5.2)	190 (2.6)	
Lymph node status				<0.001
pN0	1,296 (17.8) 1296 (17.8)	1,061 (14.6)	235 (3.2)	
pNx	5,317 (73.1)	4,635 (63.7)	682 (9.4)	
pN1–3	665 (9.1)	324 (4.5)	341 (4.7)	
Year of surgery				
2004–2007	2,424 (33.3)	2,084 (28.6)	340 (4.7)	<0.001
2008–2011	2,451 (33.7)	2,023 (27.8)	428 (5.9)	
2012–2015	2,403 (33.0)	1,913 (26.3)	490 (6.7)	

* *With percentages in parentheses. Fisher's exact test or χ^2^ test, except ^§^Student's t-test*.

### Trends in Perioperative Chemotherapy

Patients were categorized into two groups according to chemotherapy recode: 6,020 (82.7%) patients in the no/unknown group and 1,258 patients (17.3%) in the yes group. When comparing the characteristics of the two groups, we observed that patients who received perioperative chemotherapy were younger than the no/unknown group (67.6 ± 10.1 vs. 72.8 ± 10.7 years old, *P* < 0.001). In addition, perioperative chemotherapy was frequently performed in non-white race, married, higher tumor grade, advanced tumor stage, and lymph node stage patients (all *P* < 0.001) (Table 1). Moreover, chemotherapy rate was increasing significantly from 2004 to 2015 (*P* < 0.001) (Table 1).

### Survival Analyses According to Chemotherapy Recode

The 5-year and 10-year OS and CSS rates for all pT stages patients according to chemotherapy recode are shown in Table 2. For the chemotherapy group vs. the no/unknown chemotherapy group, the 5-year OS rates and CSS rates were 38.6 vs. 47.0% and 58.2 vs. 74.9%. When stratifying according to pathological tumor stage, 5-year OS rates for chemotherapy vs. no/unknown chemotherapy group were 54.5 vs. 65.1% for pT1, 46.0 vs. 48.4% for pT2, 41.2 vs. 34.1% for pT3, and 10.8 vs. 14.6% for pT4 disease. In addition, the 5-year CSS rates for the same tumor stages were 76.6 vs. 89.3%, 75.1 vs. 78.2%, 59.3 vs. 62.1% and 22.0 vs. 37.5%, respectively.

**Table 2:** Life of the 5-and 10-year overall survival and cancer-specific survival rates.

	**Overall survival rate (%)**		**Cancer-specific survival rate (%)**	
	**5 years**	**10 years**	**5 years**	**10 years**
All stages				
Chemotherapy				
Yes	38.6	26.3	58.2	51.6
No/unknown	47.0	28.9	74.9	68.8
Lymph node stage				
pN0	52.0	32.5	76.8	71.9
pNx	47.1	29.2	74.3	67.8
pN1–3	20.7	13.8	40.1	34.2
pT1 stage				
Chemotherapy				
Yes	54.5	40.5	76.6	64.6
No/unknown	65.1	42.3	89.3	84.5
Lymph node stage				
pN0	69.3	47.8	90.3	89.5
pNx	64.5	41.5	88.9	83.1
pN1–3	44.1	34.7	64.7	58.2
pT2 stage				
Chemotherapy				
Yes	46.0	29.1	75.1	68.3
No/unknown	48.4	27.3	78.2	69.3
Lymph node stage				
pN0	56.5	29.8	80.6	72.3
pNx	47.5	27.6	78.6	69.9
pN1–3	22.9	16.3	51.6	42.9
pT3 stage				
Chemotherapy				
Yes	41.2	28.3	59.3	53.8
No/unknown	34.1	20.2	62.1	54.8
Lymph node stage				
pN0	43.8	26.6	69.3	63.3
pNx	36.2	22.1	62.4	55.6
pN1–3	22.2	14.6	42.1	35.2
PT4 stage				
Chemotherapy				
Yes	10.8	5.5	22.0	18.8
No/unknown	14.6	6.3	37.5	34.6
Lymph node stage				
pN0	21.6	14.8	48.1	NA
pNx	12.8	4.8	31.2	28.8
pN1–3	9.9	4.5	19.6	NA

**Figure 1:**
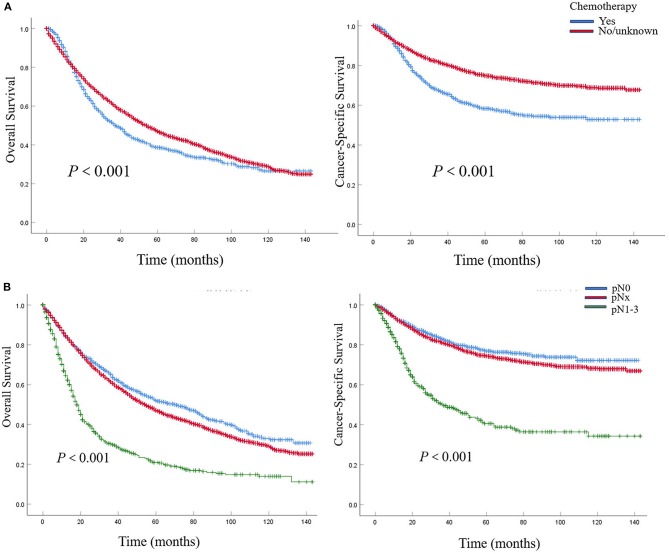
Kaplan–Meier plots depicting overall survival (OS) and cancer-specific survival (CSS), after stratification according to perioperative chemotherapy record **(A)** and lymph node stage **(B)** in 7,278 patients treated with nephroureterectomy between 2004 and 2015, within the Surveillance, Epidemiology and End Results (SEER) database.

**Figure 2:**
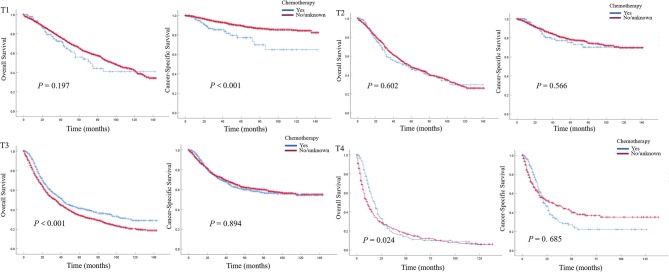
Kaplan–Meier plots depicting overall survival (OS) and cancer-specific survival (CSS) for pT1, pT2, pT3, and pT4 diseases, after stratification according to perioperative chemotherapy record in 7,278 patients treated with nephroureterectomy between 2004 and 2015, within the Surveillance, Epidemiology and End Results database.

**Figure 3:**
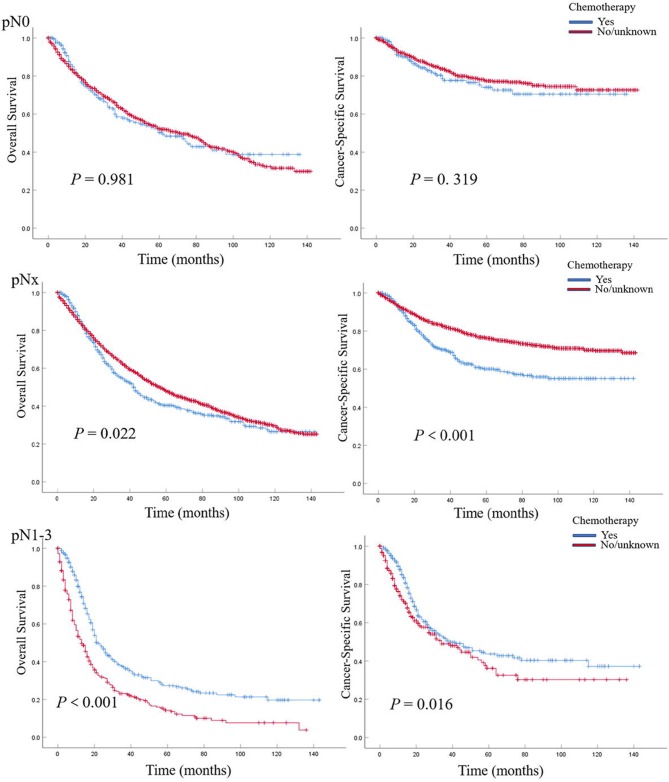
Kaplan–Meier plots depicting overall survival (OS) and cancer-specific survival (CSS) for pN0, pNx, and pN1–3 diseases, after stratification according to perioperative chemotherapy record in 7,278 patients treated with nephroureterectomy between 2004 and 2015, within the Surveillance, Epidemiology, and End Results database.

In patients with pN0 vs. pNx vs. pN1-3 disease, the 5-year OS rates were 52.0 vs. 47.1 vs. 20.7%, and the CSS rates were 76.8 vs. 74.3 vs. 40.1%, respectively. The detrimental effect of pNx and of LNM (pN1-3) for both OS and CSS was consistent across all tumor stages. Kaplan–Meier plots describing OS and CSS rates, after stratifying according to the perioperative chemotherapy record and lymph node stage are shown in Figures 1A,B. In addition, Kaplan–Meier plots depicting OS and CSS for stage-specific disease stratifying tumor stage and lymph node stage are shown in Figures 2, 3, respectively.

In multivariable COX regression analyses, patients who underwent perioperative chemotherapy had lower hazard ratio (HR) for OS (HR 0.93) but higher HR for CSS (HR 1.05) rates relative to the no/unknown group (Tables 3, 4), while both of them did not achieve statistical significance. After stratifying according to tumor stage, the beneficial effect of chemotherapy on OS was only observed in patients with pT3 (HR 0.83, *P* < 0.01) or pT4 (HR 0.74, *P* < 0.01) stage disease and disappeared for patients with pT1 (HR 1.36, *P* > 0.05) stage disease. Interestingly a detrimental impact of chemotherapy was found in pT2 patients (HR 1.32, *P* < 0.05). However, no beneficial effect was seen when similar analyses were repeated in CSS. In addition, the detrimental impact of chemotherapy was observed in pT1 patients (HR 2.31, *P* < 0.001). Moreover, the OS rate was 1.2-fold and 2.1-fold higher in patients with pN0 relative to patients with, respectively, pNx and pN1–3 stage disease (both *P* < 0.001) (Tables 3, 4). These results were consistent when analyses were repeated across all tumor stages. In addition, similar findings were found when CSS rates were tested. The results of multivariable Cox regression analyses predicting overall survival and cancer-specific survival stratified by lymph node stage are shown in Table 5. The protective effect of chemotherapy on both OS (HR 0.58, *P* < 0.001) and CSS (HR 0.70, *P* < 0.01) was observed in LNM (pN1–3) disease (Table 5).

**Table 3:** Multivariable Cox regression analyses predicting overall survival stratified by T stage.

	**All stages**	**T1**	**T2**	**T3**	**T4**
	**Multivariable^†^ HR (95% CI)**	**Multivariable^‡^ HR (95% CI)**	**Multivariable^‡^ HR (95% CI)**	**Multivariable^‡^ HR (95% CI)**	**Multivariable^‡^ HR (95% CI)**
Chemotherapy					
No/unknown	1.00 (Ref.)	1.00 (Ref.)	1.00 (Ref.)	1.00 (Ref.)	1.00 (Ref.)
Yes	0.93 (0.85–1.02)	1.36 (0.99–1.85)	1.32 (1.02–1.72)^*^	0.83 (0.74–0.94)*^*^	0.74 (0.60–0.92)*^*^
Lymph node stage					
pN0	1.00 (Ref.)	1.00 (Ref.)	1.00 (Ref.)	1.00 (Ref.)	1.00 (Ref.)
pNx	1.19 (1.09–1.31)^***^	1.06 (0.86–1.29)	1.19 (0.97–1.47)	1.15 (1.01–1.32)^*^	1.58 (1.19–2.10)*^*^
pN1–3	2.11 (1.86–2.39)^***^	2.29 (1.48–3.55)^***^	2.45 (1.69–3.55)^***^	1.87 (1.58–2.22)^***^	2.10 (1.54–2.87)^***^

* P < 0.05,

** P < 0.01,

*** P < 0.001.

† Adjusted to age, gender, race, tumor location, laterality, tumor size, tumor stage, lymph node stage, tumor grade, and year of surgery.

‡ *Adjusted to age, gender, race, tumor location, laterality, tumor size, lymph node stage, tumor grade, and year of surgery. HR, hazard ratio; 95% CI, 95% confidence interval*.

**Table 4:** Multivariable Cox regression analyses predicting cancer-specific survival stratified by T stage.

	**All stage**	**T1**	**T2**	**T3**	**T4**
	**Multivariable^†^ HR (95% CI)**	**Multivariable^‡^ HR (95% CI)**	**Multivariable^‡^ HR (95% CI)**	**Multivariable^‡^ HR (95% CI)**	**Multivariable^‡^ HR (95% CI)**
Chemotherapy					
No/Unknown	1.00 (Ref.)	1.00 (Ref.)	1.00 (Ref.)	1.00 (Ref.)	1.00 (Ref.)
Yes	1.05 (0.92–1.20)	2.31 (1.46–3.65)***	1.08 (0.69–1.67)	0.99 (0.85–1.18)	0.89 (0.67–1.19)
Lymph node stage					
pN0	1.00 (Ref.)	1.00 (Ref.)	1.00 (Ref.)	1.00 (Ref.)	1.00 (Ref.)
pNx	1.31 (1.12–1.51)***	1.19 (0.78–1.81)	1.20 (0.83–1.73)	1.23 (1.01–1.50)*	2.05 (1.36–3.10)**
pN1–3	2.52 (2.09–3.03)***	5.22 (2.61–10.44)***	3.53 (2.00–6.22)***	2.35 (1.84–3.00)***	2.60 (1.67–4.06)***

*P < 0.05, **P < 0.01, ***P < 0.001. ^†^Adjusted to age, gender, race, tumor location, laterality, tumor size, tumor stage, lymph node stage, tumor grade, and year of surgery. Adjusted to age, gender, race, tumor location, laterality, tumor size, lymph node stage, tumor grade, and year of surgery. HR, hazard ratio; 95% CI, 95% confidence interval.

**Table 5:** Multivariable Cox regression analyses predicting overall survival and cancer-specific survival stratified by lymph node stage.

	**All stage**	**N0**	**Nx**	**N1-3**
	**Multivariable^†^ HR (95% CI)**	**Multivariable^‡^ HR (95% CI)**	**Multivariable^‡^ HR (95% CI)**	**Multivariable^‡^ HR (95% CI)**
OS				
Chemotherapy				
No/Unknown	1.00 (Ref.)	1.00 (Ref.)	1.00 (Ref.)	1.00 (Ref.)
Yes	0.93 (0.85–1.02)	0.99 (0.79–1.26)	0.99 (0.88–1.12)	0.58 (0.47–0.70)***
CSS				
Chemotherapy				
No/unknown	1.00 (Ref.)	1.00 (Ref.)	1.00 (Ref.)	1.00 (Ref.)
Yes	1.05 (0.92–1.20)	0.96 (0.67–1.37)	1.16 (0.98–1.37)	0.70 (0.54–0.91)**

**P < 0.01, ***P < 0.001. ^†^Adjusted to age, gender, race, tumor location, laterality, tumor size, tumor stage, lymph node stage, tumor grade, and year of surgery. ^‡^Adjusted to age, gender, race, tumor location, laterality, tumor size, tumor grade, and year of surgery. HR, hazard ratio; 95% CI, 95% confidence interval; OS, overall survival; CSS, cancer-specific survival.

## Discussion

In this study, perioperative chemotherapy was performed in only 17.3% of the UTUC patients. However, perioperative chemotherapy was increasingly performed for patients undergoing NU year by year from 2004 to 2015, which indicated that urologists were increasingly aware of the crucial clinical role of chemotherapy. In addition, plenty of efforts have been spent for the development of an effective perioperative chemotherapy treatment to improve survival and lower recurrences. Low age, non-white race, married status, higher grade, advanced tumor, and lymph node stage were important factors contributing to the decision making of perioperative chemotherapy. This might result from the selection bias that is inherent to the retrospective nature shared by all reports including the aforesaid meta-analysis (10). On the one hand, individuals undergoing perioperative chemotherapy were likely to be those with the good general condition and renal function. On the other hand, the patients receiving chemotherapy may be those with more aggressive pathologic characteristics (11).

Previous studies have reported the effect of perioperative chemotherapy on the survival of operated UTUC patients. A meta-analysis performed by Leow et al. showed that cisplatin-based AC might bring an OS and disease-free survival (DFS) for UTUC patients (10). Seisen et al. reported an evident OS benefit in patients undergoing AC for pT3/T4 and/or LNM UTUC compared to observation following NU (11). A recent cohort demonstrated that AC could significantly improve recurrence-free survival (RFS) not OS or CSS in patients with pT3NanyM0 (12). A National Cancer Database (NCBD)-based study showed that preoperative chemotherapy evidently benefited OS for patients with LNM (13). In the Kaplan–Meier plots of this current study, a trend toward better OS or CSS for no/unknown chemotherapy group was observed in both all-stage population and pNx population, which may be due to the selection bias mentioned above. Interestingly, an evident OS benefit in patients who underwent perioperative chemotherapy was seen in pT3/T4 and LNM patients. In addition, a significant CSS benefit was observed in LNM. In addition, this finding is consistent with the previous study (11, 14). To further investigate the stage-specific impact of perioperative chemotherapy on survival in UTUC patients, we performed Cox regression analyses to mitigate potential selection bias. Indeed, perioperative chemotherapy improved OS in patients with pT3/T4 and LNM and CSS in patients solely with LNM, which is consistent with the results of Kaplan–Meier plots aforementioned. Similarly, POUT trial, an ongoing randomized controlled trial of adjuvant chemotherapy vs. surveillance in patients with pT2-T4N0-3M0 UTUC following NU, demonstrated that a significant 2-year disease-free survival (DFS) benefit was observed in patients treated with chemotherapy (70 vs. 51%), which supports the use of adjuvant chemotherapy as a new standard of care in these high-risk UTUC patients (15). Based on these findings, we could speculate that perioperative chemotherapy should be taken into account for pT3/4 or LNM UTUC patients with good tolerance. However, we observed that chemotherapy has a detrimental effect on OS in patients with pT2 and CSS in patients with pT1, which indicated that perioperative chemotherapy should be carefully deliberated for these organ-confined UTUC patients, even with better health conditions. There might be a balance between the advantages (killing cancer cells) and disadvantages (comorbidities and impaired renal function after RNU) of perioperative chemotherapy. For pT1/2 UTUC patients, the disadvantages might play the main part especially in those with poor health conditions. Thus, comorbidities and impaired renal function after RNU (16) may play a role in reducing survival in these patients.

Several limitations of this study should be acknowledged. First, these findings should be interpreted within the limitations of the retrospective study design including selection bias. Thus, the results require further randomized clinical trials. Second, the two groups might differ in recorded and unrecorded variables because patients were not randomized to receive chemotherapy or not. In addition, detailed information on perioperative chemotherapy, such as when to perform chemotherapy and what regimen to select, is not available in SEER. In addition, the no/unknown group included patients with unclear chemotherapy information. All of these may affect survival. Third, SEER provides no information on the renal function and comorbidities, so we cannot fully control for granular performance status differences between the two groups.

To summarize, we found that perioperative chemotherapy was more frequently performed in locally advanced UTUC patients. The beneficial effect of chemotherapy on OS was evident in pT3/pT4 and pN+ patients. In addition, a clear CSS benefit was observed in patients who received chemotherapy for pN+ UTUC, while perioperative chemotherapy may reduce CSS for pT1 and OS for pT2 patients following NU. Although the common biases related to the observational study design limited our results, we believe that these findings should be considered when advising perioperative chemotherapy management of UTUC.

## Data Availability Statement

Publicly available datasets were analyzed in this study. This data can be found here: Surveillance, Epidemiology, and End Results (SEER) Program (www.seer.cancer.gov) SEER^*^Stat Database.

## Ethics Statement

For the institutional cohorts, data were extracted from the Surveillance, Epidemiology, and End Results database. This article does not contain any studies with human participants performed by any of the authors.

## Author Contributions

All authors contributed toward data analysis, drafting and writing the paper, gave final approval of the version to be submitted, and agreed to be accountable for all aspects of the work.

## Conflict of Interest

The authors declare that the research was conducted in the absence of any commercial or financial relationships that could be construed as a potential conflict of interest.

## Abbreviations

UTUC: upper urinary tract urothelial carcinoma
NU: nephroureterectomy
LND: lymph node dissection
LNM: lymph node metastasis
OS: overall survival
CSS: cancer-specific survival
NC: neoadjuvant chemotherapy
AC: adjuvant chemotherapy.
